# Extended-spectrum beta-lactamases found in *Escherichia coli* isolates obtained from blood cultures and corresponding stool specimen

**DOI:** 10.1038/s41598-023-36240-y

**Published:** 2023-06-02

**Authors:** Nina Doerr, Nadine Dietze, Norman Lippmann, Arne C. Rodloff

**Affiliations:** Institute Medical Microbiology and Virology, Microbiology Department, Leipzig University, Johannisallee 30, 04103 Leipzig, Germany

**Keywords:** Antimicrobial resistance, Epidemiology, Health care, Microbiology, Bacteria

## Abstract

With extended-spectrum β-lactamases (ESBLs) and CTX-M enzymes being on the rise, antimicrobial treatment of enterobacterial infections is becoming more and more challenging. Our study aimed at a molecular characterization of phenotypically ESBL-positive *E. coli* strains obtained from blood cultures of patients of the University Hospital of Leipzig (UKL), Germany. The presence of CMY-2, CTX-M-14 and CTX-M-15 was investigated using Streck ARM-D Kit (Streck, USA). Real-time amplifications were performed by QIAGEN Rotor-Gene Q MDx Thermocycler (QIAGEN, Thermo Fisher Scientific, USA). Antibiograms as well as epidemiological data were evaluated. Among 117 cases, 74.4% of the isolates showed a resistance to ciprofloxacin, piperacillin and ceftazidime or cefotaxime while being susceptible to imipenem/meropenem. The proportion of ciprofloxacin resistance was significantly higher than the proportion of ciprofloxacin susceptibility. At least one of the investigated genes was detected in 93.1% of the blood culture *E. coli* isolates: CTX-M-15 (66.7%), CTX-M-14 (25.6%) or the plasmid-mediated ampC gene CMY-2 (3.4%). 2.6% were tested positive for two resistance genes. 94 of the corresponding stool specimens tested positive for ESBL producing *E. coli* (94/112, 83.9%). 79 (79/94, 84%) *E. coli* strains found in the stool samples matched with the respective patient’s blood culture isolate phenotypically (MALDI-TOF, antibiogram). The distribution of resistance genes was in accordance with recent studies in Germany as well as worldwide. This study provides indications of an endogenous focus of infection and emphasize the importance of screening programs for high-risk patients.

## Introduction

*Escherichia coli* is known to be a part of the natural human intestinal flora but can also cause intestinal or extraintestinal infections^1^. In particular, *E. coli* is a frequent isolate in adult patients with bacteremia^2^. The most common sources for *E. coli* bloodstream infections are urinary tract infections, abdominal sepsis and pneumonia^3,4^.

Antimicrobial treatment of such infections is nowadays complicated by the fact that the prevalence of *Enterobacteriales* producing extended-spectrum β-lactamases (ESBLs) that came to attention first in the 1980s is on the rise ever since^5^. The first extended-spectrum β-lactamases described were of the TEM- and SHV-type and were primarily isolated from *E. coli* and *K. pneumoniae*^6^. CTX-M-Group ESBLs were first isolated from *E. coli* and reported in Germany in 1989^7^. By now, they have become the most common ESBLs worldwide with CTX-M-15 and CTX-M-14 enzymes being most frequently found in human specimens globally^8,9^.

In this context and considering the rising antimicrobial resistance of *E. coli* due to extended-spectrum β-lactamases, this study aimed at the molecular characterization of ESBLs detected in *E. coli* strains obtained from blood cultures of patients of the University Hospital of Leipzig (UKL), Germany. Furthermore, our study provides epidemiological data and resistance patterns of the isolated strains. In order to find indications of an endogenous focus of infection for the *E. coli* bacteremia, the laboratory database was searched for corresponding stool samples with evidence of ESBL producing *E. coli* for each patient. Whenever available, resistance patterns from both strains (blood culture and stool sample) of the individual patients were compared.

## Materials and methods

### *E. coli* strains

The *E. coli* strains investigated with this study were all collected between 2015 and 2018 from patients of the UKL. They were either blood culture isolates or strains that were recovered during a continuous risk-adapted screening of stool samples for multiple drug resistant strains.

As part of this screening, stool samples and rectal swabs are examined for the presence of carbapenemase- and β-lactamase-producing bacteria employing the chromogenic media CHROMagar ESBL and CHROMagarTM ESBL (Mast Diagnostica, Germany) as initial test procedure. The stool and rectal samples were collected as part of this UKL screening program and were neither collected by any of the authors nor for the purposes of this study in the first place. Therefore, there were no human participants involved in this study. All strains recovered were identified with MALDI-TOF (BioMerieux, France). Altogether, 117 strains from blood cultures (ECB) with phenotypic evidence of ESBL production and 112 strains recovered from screening (ECS) were considered for further analysis. For 94 of the bacteremic patients ESBL positive stool screening cultures were available. Those as well as all blood culture isolates were further analyzed with this study.

### Susceptibility test

All *E. coli* isolates were submitted to broth microdilution performed according to the European Committee on Antimicrobial Susceptibility Testing (EUCAST). Minimum inhibitory concentrations (MICs) for the following antibiotics were determined: ampicillin, ampicillin/sulbactam, piperacillin, piperacillin/tazobactam, ceftazidime, cefotaxime, cefuroxime, aztreonam, imipenem, meropenem, amikacin, gentamicin, tobramycin, ciprofloxacin, levofloxacin, moxifloxacin, colistin, fosfomycin, trimethoprim/sulfamethoxazole and tigecycline. *E. coli* ATCC 25922 was used as quality control strain.

### ESBL detection

Phenotypic testing for ESBL production was performed for all isolates with a MIC of ≥ 1 mg/l for either cefotaxime, ceftazidime or aztreonam. For this purpose, a gradient test system using cefotaxime/clavulanic acid and ceftazidime/clavulanic acid test strips (Etest®, bioMérieux, France) was employed. According to the recommendations of the manufacturer, individual bacterial colonies were transferred to 0.5% NaCl solution until a MC Farland standard of 0.5 was obtained. The suspension was then streaked onto Müller-Hinton agar, the strips were applied, and the tests incubated for 18 to 24 h at 37 °C. All isolates positive in the gradient test were then analyzed for the presence of extended-spectrum beta-lactamase genes CTX-M-14 and CTX-M-15 and the plasmid-mediated ampC gene CMY-2.

### ESBL gene detection

Single colonies of individual *E. coli* isolates were suspended in sterile Protease-, RNase-, and DNase-free water and then submitted to automated DNA extraction by the MagNa Pure 96 system (Roche Diagnostics, France). For the purpose of identifying the target genes (CMY-2, CTX-M-14 und CTX-M-15), the commercial PCR Mix 1 of Streck ARM-D® Kit (Streck, USA), β-Lactamase + MasterMix with fluorescence-marked DNA-probes was used. Real-time amplifications of the target genes were performed by QIAGEN Rotor-Gene Q MDx Thermocycler (QIAGEN, Thermo Fisher Scientific, USA) in accordance with the manufacturer’s instructions.

### Data collection and evaluation

Antibiograms and epidemiological data were collected by laboratory IT Hybase (epiNET AG, Germany) and LabCenter (i-solutions health GmbH, Germany). HyBase® as well as Microsoft Excel were used for the following evaluations.

## Results

In total, 130 ESBL producing *E. coli* isolates were preserved and included in the further investigation. Since 13 isolates turned out to be duplicates of the same patient, those were not included in any statistical evaluation leaving 117 isolates for further analysis.

### ECB-data

During the time period of 2015 to 2018, 159,074 blood cultures were submitted for microbiological evaluation for patients of the UKL. Out of these, 26,427 yielded bacterial growths (positivity rate: 16.6%). Since multiple cultures were submitted for individual patients with positive culture (n = 9910), a corresponding diagnosis of bacteremia was made in 6.2% of all cases. Altogether, in 1020 cases a bacteremia with *E. coli* was documented (10.3%) including 153 cases where ESBL producing strains were detected (15%). Thus, out of 9910 patients suffering from bacteremia, ESBL producing *E. coli* strains were documented in 1.5% of the cases. In total, 130 ESBL producing *E. coli* isolates were collected and included in the investigations. Among these, 13 isolates were duplicates of individual patients, so they were eliminated from any statistical evaluation leaving 117 isolates for further interpretation.

**Figure 1:**
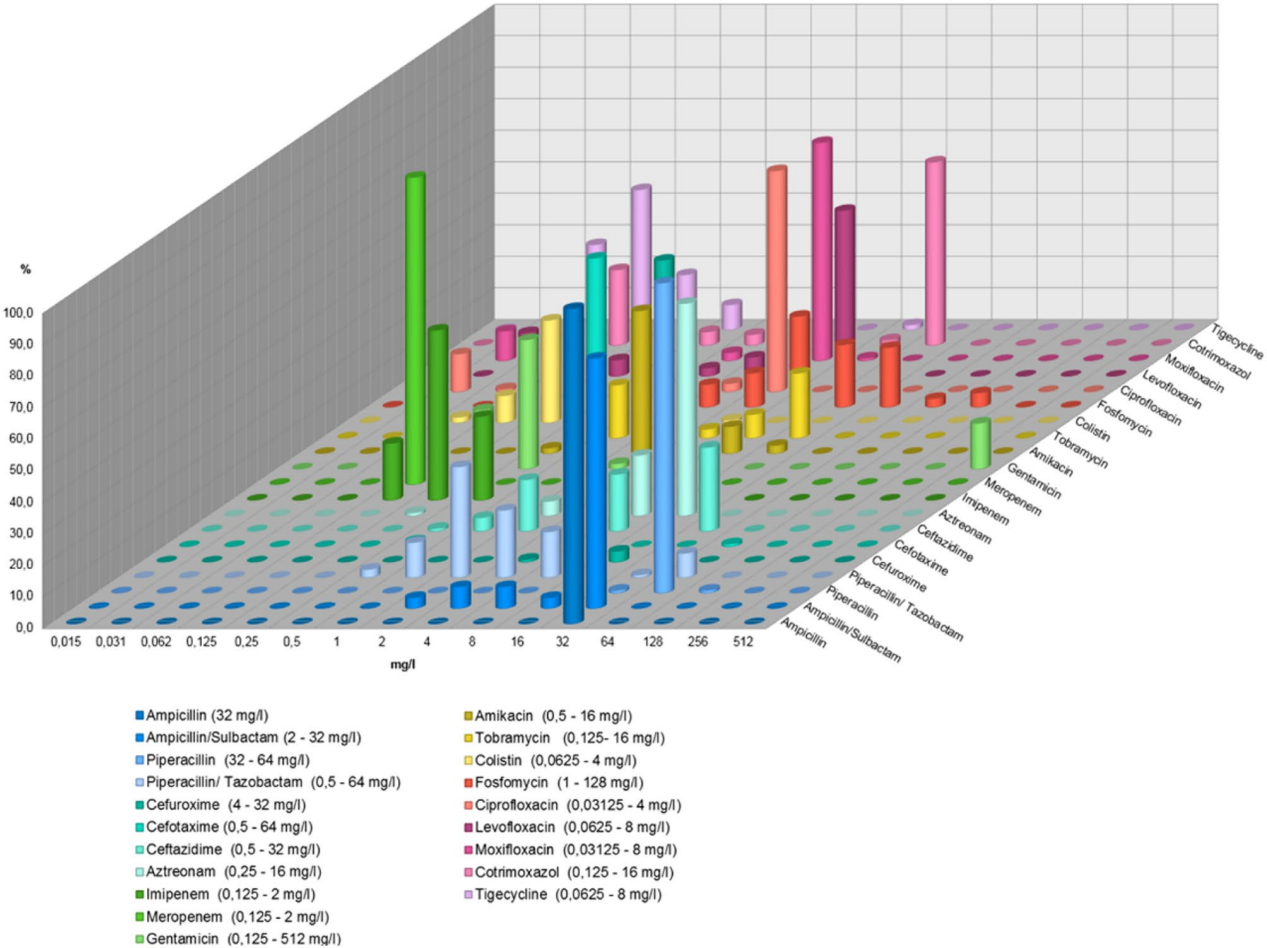
MIC distributions of ECB.

Susceptibility data for the 117 ECBs are given with Table 1 and MIC distributions are depicted in Fig. 1. The results show that aside from the ß-lactam resistance, 74.4% (87/117) were also resistant to ciprofloxacin.

**Table 1:** Antimicrobial susceptibility of ECB.

Antimicrobial agent	Susceptible	Intermediate	Resistant
Ampicillin	0/116 (0%)	0/116 (0%)	116/116 (100%)
Ampicillin/sulbactam	3/116 (2.6%)	17/116 (14.7%)	96/116 (82.8%)
Piperacillin	0/117 (0%)	0/117 (0%)	117/117 (100%)
Piperacillin/tazobactam	95/117 (81.2%)	7/117 (6.0%)	15/117 (12.8%)
Cefuroxime	0/116 (0%)	1/116 (0.9%)	115/116 (99.1%)
Cefotaxime	3/117 (2.6%)	2/117 (1.7%)	112/117 (95.7%)
Ceftazidime	6/117 (5.1%)	38/1117 (32.5%)	73/117 (62.4%)
Aztreonam	2/110 (1.8%)	12/110 (10.9%)	96/110 (87.3%)
Imipenem	117/117 (100%)	0/117 (0%)	0/117 (0%)
Meropenem	117/117 (100%)	0/117 (0%)	0/117 (0%)
Gentamicin	97/117 (82.9%)	1/117 (0.9%)	19/117 (16.2%)
Amikacin	114/117 (97.4%)	3/117 (2.6%)	0/117 (0%)
Tobramycin	74/107 (69.2%)	3/107 (2.8%)	30/107 (28.0%)
Colistin	116/117 (99.1%)	0/117 (0%)	1/117 (0.9%)
Fosfomycin	103/111 (92.8%)	0/111 (0%)	8/111 (7.2%)
Ciprofloxacin	28/117 (23.9%)	2/117 (1.7%)	87/117 (74.4%)
Levofloxacin	29/116 (26.7%)	1/116 (5.2%)	79/116 (68.1%)
Moxifloxacin	29/117 (24.8%)	1/117 (0.9%)	87/117 (74.4%)
Cotrimoxazol	46/117 (39.3%)	2/117 (1.7%)	69/117 (59.0%)
Tigecycline	62/63 (98.4%)	0/63 (0%)	1/63 (1.6%)

**Figure 2:**
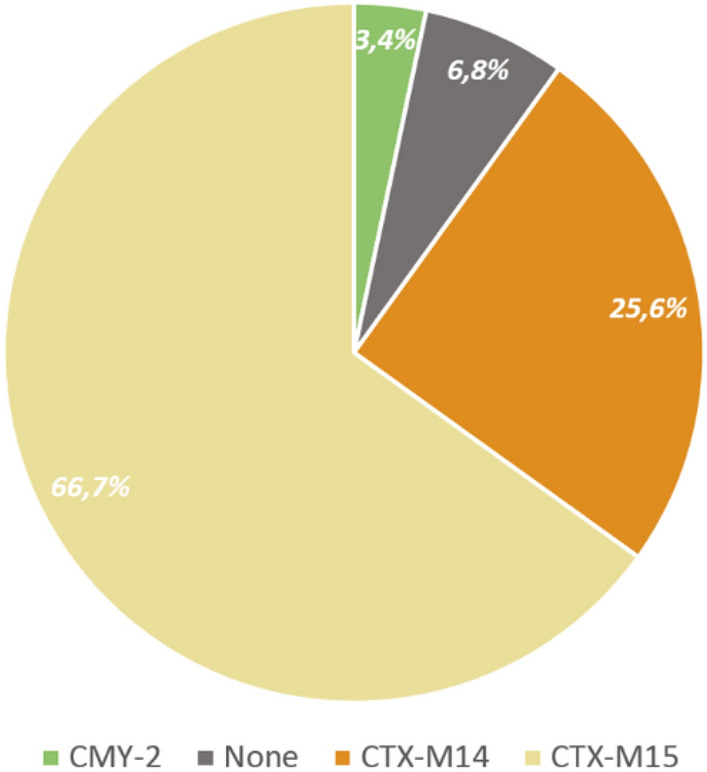
Distribution of detected antimicrobial resistance genes (ESBL producing and plasmid-mediated ampC genes).

In 109 out of the 117 isolates (93.1%), there was at least one of the investigated genes detected, genotype CTX-M-15 being the most prevalent (78/117, 66.7%), followed by genotype CTX-M-14 (30/117, 25.6%) and CMY-2 (4/117, 3.4%). Three isolates (2.6%) were tested positive for the possession of two resistance genes simultaneously (one for CMY-2 and CTX-M-15, one for CTX-M-14 and CMY-2, one for CTX-M-15 and CTX-M-14). Finally, 8 isolates (8/117, 6.8%) were negative for the tested genes. The distribution of the examined genes is shown in Fig. 2.

Susceptibility testing for CTX-M-15-positive isolates showed that > 97% of the samples (76/78) were resistant to cefotaxime, whereas only two of the tested isolates were susceptible to cefotaxime (2/78, 2.6%). There was a resistance to ceftazidime in > 61% of the CTX-M-15-positive isolates (48/78).

All CTX-M-14-positive isolates (30/30) were resistant to cefotaxime with a MIC > 4 mg/l, whereas 60% were also not susceptible to ceftazidime (18/30). Beside a resistance to ß-lactam antibiotics, a high proportion of strains with an increased ciprofloxacin MIC level (≥ 0.5 mg/l) was detected (89/117, 76.1%), even 74.4% (87/117) with a MIC Level ≥ 1 mg/l.

### ECS-data

Of the 117 bacteremic patients included in this study, 112 had submitted stool samples prior to the time of bacteremia. 94 of these specimens tested positive for ESBL producing *E. coli* (94/112, 83.9%). Retrospectively, a total of 79 (79/94, 84%) *E. coli* strains found in the stool samples matched with the respective patient’s blood culture isolate phenotypically (MALDI-TOF, antibiogram). The comparison between the stool samples and blood cultures that tested positive for ESBL is shown in Table 2. Considering the ciprofloxacin resistance, 55.4% (62/112) of the patients were colonized with an ESBL positive and ciprofloxacin resistant strain. 15 (15/94, 16%) of the isolates showed no phenotypic match of blood culture isolate and respective stool sample.

**Table 2:** Comparison between ESBL positive stool samples and blood cultures.

ESBL positive stool samples	Stool samples resistant to ciprofloxacin	Simultaneous blood culture and stool sample	Matches resistant to ciprofloxacin	Matches susceptible to ciprofloxacin
94	67/94 (72.3%)	79/94 (84.0%)	62/94 (66.0%)	17/94 (18.1%)

### Infection due to ESBL-*E. coli*

Table 3 shows the epidemiology of multi-resistant *E. coli* during the investigation period. Between 2015 and 2018 there were 12,346 cases of infection by invasive *E. coli,* id est isolations of *E. coli* excluding rectal swabs and stool samples. Overall, 13.1% (1614/12,346) were caused by ESBL-producing isolates, while 8.0% (988/12,346) of these were caused by ESBL-producing strains with additional ciprofloxacin resistance; within ESBL-positive isolates, the proportion of ciprofloxacin resistance (61.2%, 988/1614) was higher than the proportion of ciprofloxacin susceptibility significantly. Further, 1020 cases of bloodstream infections by *E. coli* were detected during the investigation period. Among the 117 cases included in this study, 74.4% of the isolates were showing a resistance to ciprofloxacin, piperacillin as well as ceftazidime or cefotaxime while being susceptible to imipenem/ meropenem.

**Table 3:** *E. coli* epidemiology from 2015 to 2018.

*E. coli* (2015–2018)					
	Total	ESBL *E. coli*			
			Ciprofloxacin susceptible	Ciprofloxacin resistant	
	(n)	(n)	(n)	(n)	*p*
Invasive *E. coli* including blood cultures	12,346	1614	626	988	0.0000000000
Blood cultures *E. coli* (indicated in this study)	1020	117	30	87	0.0000000641
Corresponding stool sample	112/117	94	27	67	
Matches resistant to ciprofloxacin			–	62	
Matches susceptible to ciprofloxacin			17	–	

## Discussion

Our study focused on patients with bacteremia caused by ESBL producing *E. coli* strains. The dominant ESBL genotype in our isolates was CTX-M-15 (78/117, 66.7%), followed by genotype CTX-M-14 (30/117, 25.6%) and AmpC CMY-2 (4/117, 3.4%). These results underscore earlier reports that worldwide CTX-M-15 is the most prevalent ESBL found in *Enterobacteriaceae*^10^. They are also in line with a previous study in Germany addressing the molecular epidemiology of ESBL producing *Enterobacteriaceae* among 156 nursing home residents in Bavaria^11^. Ha YE et al. analyzed bloodstream infections due to *E. coli* in cancer patients of the Samsung Medical Center in Seoul—they found CTX-M-14 (37.7%), CTX-M-15 (26.1%) or both CTX-M-15 and CTX-M-14 (10.1%) in their isolates^12^. However, investigators at Nara Medical University, a tertiary care hospital in Japan, reporting on cases of bacteremia with ESBL producing *E. coli*, found that ESBL-EC CTX-M-27 (33.3%) and CTX-M-14 (30%) were the most prevalent genes^13^.

Several studies assessed the fecal carriage rate of ESBL producing *E. coli*, i.e. the rate was reported to be 14.7% in Bavarian nursing home residents and 6.3% in the healthy population^11^. The prevalence of ESBL producing *E. coli* in fecal samples of 650 inpatients reported in a study in China, Beijing, was 25.7%^14^, while a study from Ankara, Turkey, reported the ESBL carriage rate at 34.3% for 1402 outpatients with *E. coli* strains being the most frequent ones^15^.

Since an extensive screening program for multidrug resistant microorganisms is in place in our hospital, we were also able to address the fecal carriage of ESBL producing *E. coli* strains of the patients with bloodstream infections of our study. Interestingly, of the 117 bacteremic patients included in this study, 112 had submitted stool samples prior to time of bacteremia and 94 of these specimens yielded ESBL producing *E. coli* (94/112, 83.9%). 79 of the *E. coli* strains found in the stool samples matched with the respective patient’s blood culture isolate phenotypically, suggesting a high incidence of endogenous infections. Reddy et al. reported earlier on an infection control program addressing ESBL producing *Enterobacteriales*—they found that out of 413 colonized patients 8.5% developed a subsequent bloodstream infection, amounting to 34.3% of all bloodstream infections by ESBL producing *Enterobacteriales*^16^. Our data emphasize once more the value of risk adapted screening and—in case of an infection—the necessity to select an empiric therapy accordingly. Moreover, the epidemiology of the underlying ESBL genes should be surveyed on a regular basis and analyzed together with the respective susceptibility data.

With this study *E. coli* and three genes were addressed, only. This is an obvious shortcoming and we cannot rule out that other ESBL genes were also present in our isolates. However, in 109 out of the 117 isolates (93.1%), there was at least one of the investigated genes detected and the results are in line with the epidemiology reported globally.

## Conclusion

The molecular investigation of ESBL producing *E. coli* collected from blood cultures from patients at the University Hospital of Leipzig, Germany, between 2015 and 2018 showed a distribution of resistance genes that was in accordance with recent studies in Germany as well as worldwide with CTX-M-15 being the most prevalent genotype in our study.

The results of our study provide possible indications of an endogenous focus of infection for an *E. coli* bacteremia and, therefore, emphasize the importance of screening programs for high-risk patients.

## Data Availability

The datasets generated during and/or analyzed during the current study are available from the corresponding author on reasonable request.

## Abbreviations

ESBL: Extended-spectrum β-lactamase
ECB: *E. coli* strains isolated from blood cultures
ECS: *E. coli* strains recovered from stool sample-screening
MIC: Minimum inhibitory concentration
